# Retropupillary Iris-fixated versus Sutured Scleral-fixated Intraocular Lenses

**DOI:** 10.22336/rjo.2024.04

**Published:** 2024

**Authors:** Rozaliya Hristova, Denitsa Cholakova, Alexander Oscar, Bogumil Wowra, Dimitar Dzhelebov, Yani Zdravkov

**Affiliations:** * Department of Ophthalmology, University Hospital Alexandrovska, Medical University Sofia, Sofia, Bulgaria; ** Department of Ophthalmology, Medical University of Silesia, Katowice, Poland; *** Department of Ophthalmology, Trakia University, Stara Zagora, Bulgaria

**Keywords:** aphakia, scleral-fixated IOL, iris-fixated IOL, Artisan, endothelial cell loss

## Abstract

**Aim:** To compare the anatomical and functional results and patient satisfaction following retropupillary implantation of Artisan Aphakia iris-fixated intraocular lens (rAAIF) and sutured scleral fixated intraocular lens (SFIOL).

**Subjects and methods:** We presented a prospective double-arm non-blinded study. Forty-one eyes with acquired aphakia, no age-related macular degeneration, no previous keratoplasty, no combined procedures, no AC reaction (cells, fibrin), normal intraocular pressure, no history of endothelial corneal dystrophy in relatives or fellow eye were included. Indications, complications, corrected distance visual acuity (CDVA), endothelial cell density (ECD), and patient satisfaction score were assessed.

**Results:** Retropupillary AAIF was implanted in 21 (51.22%) eyes and SFIOL in 20 (48.78%) eyes. The most common indication was complicated cataract surgery in 18 cases (43.90%), followed by trauma in 16 (39.02%), and spontaneous dislocation in 7 (17.07%). No difference between rAAIF and SFIOL in terms of sex, laterality (χ=0.13, *p*=0.72), indications (χ=0.78, *p*=0.68), previous ocular history, and comorbidities was observed. The complications and the visual outcomes at 6 months postoperatively were similar between the two groups (*p*=0.95 and *p*=0.321, respectively). The ECD loss in the two groups was also similar (*p*=0.89). The patient satisfaction score was 58.67±8.80 in the rAAIF and 56.69±11.50 in the SFIOL group, which was statistically similar (*p*=0.764).

**Conclusion:** Retropupillary AAIF and SFIOL showed similar results concerning visual acuity, endothelial cell loss, and patient satisfaction. Careful preoperative individual assessment is required to have optimal results with either technique.

**Abbreviations:** AAIF = Artisan Aphakia iris-fixated intraocular lens, rAAIF = retropupillary Artisan Aphakia iris-fixated intraocular lens, CDVA = corrected distance visual acuity, ECD = endothelial cell density, IOL = intraocular lens, SD = standard deviation, SFIOL = scleral fixated intraocular lens

## Introduction

Cataract surgery is one of the most common surgical procedures worldwide - 10 million cataract operations are performed each year in the world [**1**]. The big operating volume, equipment improvement, accessible surgical training, and fast learning curve have made cataract surgery safe with a complication rate of around 0.1% [**2**]. However, a possible sight-threatening complication is surgical aphakia. Without sufficient structural support for placing a posterior chamber IOL in the capsular bag, aphakia could be corrected in several ways - angle-supported, iris-fixated, and scleral-fixated intraocular lens (IOL). 

Iris-supported lenses such as the Artisan Aphakia Iris Fixation IOL (AAIF) could be implanted both in the anterior and posterior chamber with growing evidence that retropupillary implantation is superior concerning visual acuity, endothelial cell density (ECD), and complication rates. The surgical technique for AAIF IOL implantation is more time effective compared to other approaches for correcting aphakia as well. Nevertheless, both anterior and retropupillary implantation of AAIF require big incisions with surgically induced astigmatism, anterior vitrectomy, and usually a peripheral iridotomy [**3**]. 

Scleral-fixated IOLs (SFIOL) offer better predictability of refractive results, are closer to physiologically effective lens position, have better ECD, and cause less surgically induced astigmatism. However, the implantation requires anterior vitrectomy, sutures, and complex surgical skills. It is also more time-consuming compared to AAIF [**4**]. 

In our study, we compared the visual performance, endothelial cell changes as well as patient satisfaction between retropupillary AAIF and SFIOL.

## Subjects and methods

This was a prospective double-arm non-blinded study on the clinical results following retropupillary implantation of iris-fixated IOL and SFIOL conducted from May 2021 to May 2023, and approved by the Ethical Committee of Medical University Sofia. All patients signed an informed consent before participation in the study. All procedures involving human participants were performed following the 1964 Helsinki Declaration and its later amendments or comparable ethical standards.

All patients underwent a complete ophthalmological examination, including visual acuity, tonometry, and optical coherence tomography to detect preexisting macular changes and postoperative complications, mainly cystoid macular edema. Inclusion criteria were: patients with surgically induced acquired aphakia with secondary implantation of retropupillary iris-fixated IOL or SFIOL, no age-related macular degeneration, no previous keratoplasty and no combined procedures, no AC reaction (cells, fibrin) at the time of secondary implantation, normal intraocular pressure (IOP) either with or without medication, no history of endothelial corneal dystrophy in the relative or fellow eye. All surgeries were performed by a single ophthalmic surgeon with more than 10 years of experience, under retrobulbar anesthesia. 

For IOL power calculations, the IOL Master Model 700 (Carl Zeiss Meditec, Dublin, CA) was used. Only scans with OK marking and SD less than 0.02 were included. The power of the lens was selected based on the SRKT and Holladay 1 formulas.

The surgical procedure for iris-fixated IOL included one corneal paracentesis, a limbal incision 5.4 mm, centered at the 12 o’clock position, and filling of AC and PC with cohesive viscoelastic material. The iris-claw IOL was then inserted in the anterior chamber, rotated with an Artisan lens forceps to a horizontal position, and centered on the pupil. The optic of the iris-claw IOL was held securely with the forceps. Next, the two haptics were gently slid behind the iris and the optic was lifted slightly forward toward the posterior surface of the iris so that the claw configuration of the haptic could be recognized from above on the anterior iris surface. With the other hand, a long micro-spatula was used through the lateral paracentesis to insert iris tissue into the claw. The second haptic was fixated in the same way, using the same spatula. Care was taken to apply gentle pressure over the slotted center of the lens haptic to enclave a fair amount of iris tissue to avoid ovalization of the pupil and to decrease the effect of enclavation on the movement of the pupil. Peripheral iridectomies were performed in all cases. Anterior vitrectomy was used as necessary. Viscoelastic was removed via irrigation and aspiration. The corneal incision was secured with 10/0 nylon interrupted sutures. 

The scleral fixation IOL technique included two corneal paracenteses, a limbal incision 2.7 mm, centered at the 12 o’clock position, creation of scleral tunnels at 2 mm from the limbus, taking care to avoid major ciliary vessels at 3 and 9 o’clock, filling of AC and PC with cohesive viscoelastic, anterior vitrectomy. The IOL (HOYA i-sert PY60AD) was implanted through the main incision (2.7 mm), supported on the iris, and was followed by mounting the haptics of the three-piece lens with 9/0 Prolene and externalization of the sutures through the scleral tunnels. The two haptics were gently slid behind the iris and careful manipulation of the sutures was used for IOL centration, followed by securely tying the sutures in the desired position and closing the scleral pockets, covered by 8/0 Prolene conjunctival sutures. Viscoelastic was removed via irrigation and aspiration. The corneal incision was secured with a single 10/0 nylon suture.

At the end of all surgeries, a prophylactic antibiotic (cefuroxime 0.01%) was injected intracamerally and dexamethasone subconjunctivally. The postoperative regimen included a topical antibiotic (Ofloxacin) and steroid drops (Dexamethasone) four times daily in the operated eye for four weeks, as well as IOP-lowering medication as needed. Follow-up exams were scheduled on day 1, day 7, and day 30, at 3 months and 6 months after the surgery. Each follow-up exam included best-corrected visual acuity, IOP, slit lamp examination, and OCT to determine the presence of CME.

The Konan specular microscope was used to measure the endothelial cell count before and after the lens implantation. We did not consider the ECD before the lens removal surgery to ascertain the loss induced only by the implantation technique.

A questionnaire based on standard IOL questionnaires for patient satisfaction was repurposed, including satisfaction with uncorrected and corrected distance and near visual acuity, night vision, visual phenomena, ocular surface complaints, etc. The reliability of the test was ascertained with Cronbach’s alpha test. The questionnaire used in this study utilized a 5-point scale with 1 being the least and 5 being the most satisfactory response. The overall satisfaction score was an arithmetical sum of the values for each question.

The main outcome measures were postoperative corrected distance visual acuity (CDVA), as assessed with Snellen charts, changes in ECD as obtained by comparing before and after measurements by the Konan specular microscope, and the score from the patient satisfaction questionnaire.

Extracted data were entered into a spreadsheet. Statistical analysis was performed using the IBM SPSS statistical package for Windows Version 26.0 (IBM Corp., Armonk, NY, USA). Data were expressed as frequency (percentage) for nominal data, mean ± standard deviation of the mean (SD). Statistical significance between the study groups regarding the previously mentioned parameters was determined using the chi-square test for categorical variables, and either Student’s t-test or Mann-Whitney test for continuous variables. Values of p under 0.05 were considered statistically significant. The sample size was confirmed retrospectively at the alpha level of 0.05 and power of analysis at 90%.

## Results

In this study, 41 eyes of 41 patients were included. Of those, 29 (70.73%) were male and 12 (29.27%) were female. The mean age of the patients was 74.38 ± 9.46 years. Of the 41 patients, the left eye was involved in 21 (51.22%). Retropupillary AAIF IOL was implanted in 21 (51.22%) eyes and SFIOL was performed in 20 (48.78%) eyes.

The most common indication for secondary implantation was complicated cataract surgery in 18 cases (43.90%), followed by trauma in 16 (39.02%) and spontaneous dislocation in 7 (17.07%). Comorbidities were discovered in 27 patients - arterial hypertension in 15 (36.59%), glaucoma in 11 (26.83%), of which PEX was present in 5 (12.2%), diabetes mellitus in 2 (4.88%), and uveitis, vitreous hemorrhage, retinal detachment, intraoperative floppy iris syndrome, Marfan syndrome and asthma in one patient for each. The mean corneal astigmatism following surgery was 1.58 ± 1.57D. Visual acuity improved from 0.12 ± 0.16 before surgery to 0.28 ± 0.23 after surgery (p<0.0001).

The mean axial length of all patients was 23.55 ± 0.81. The mean K1 was 43.12 ± 1.53, K2 - 44.71 ± 2.06. Overall IOL power was 19.08 ± 1.87. 

The most common complications were transient corneal edema in 9 (21.95%) patients and high postoperative IOP in 7 (17.07%). 

*Comparison of SFIOL and retropupillary AAIF*

No difference was observed between rAAIF and SFIOL in terms of sex (**Table 1**), laterality (χ=0.13, *p*=0.72), and indications (χ=0.78, *p*=0.68). 

**Table 1:** Comparison between age and sex in the two groups

Group	*n*	Sex		Age (a, x ± SD)
		M	F	
Artisan group	21	15	6	79.24 ± 4.37
Scleral fixation group	20	14	6	70 ± 10.32
Test value		0.01a		-3.24b
*P*		0.92		0.001

**Fig. 1:**
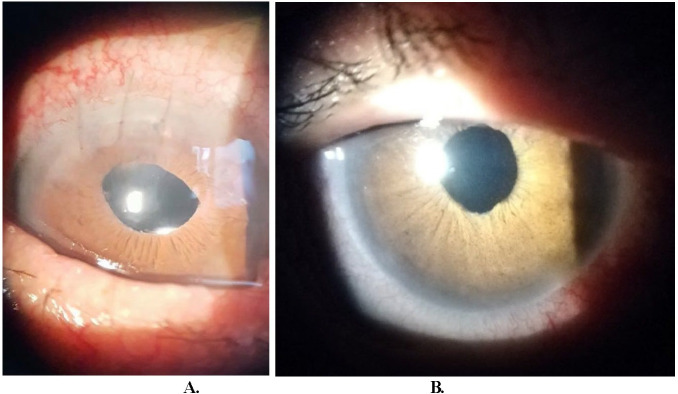
Postoperative slit-lamp examination. **A.** Retropupillary implanted Artisan Aphakia intraocular lens, the corneal wound is adapted with several 10/0 sutures. There is slight pupillary ovalization. **B.** Scleral-fixated intraocular lens after removal of the single corneal suture

Patients in the SFIOL group were younger. Complications were similar across the two groups - 5 patients with transient corneal edema in the rAAIF and 4 in the SFIOL group, high IOP in 4 patients with rAAIF, and 3 with SFIOL (χ=0.11, *p*=0.95). Pupil ovalization was only observed in the rAAIF group as expected (**Fig. 1**). A preexisting idiopathic epiretinal membrane was observed in one patient, but no cases of cystoid macular edema were detected in either group. 

**Fig. 2:**
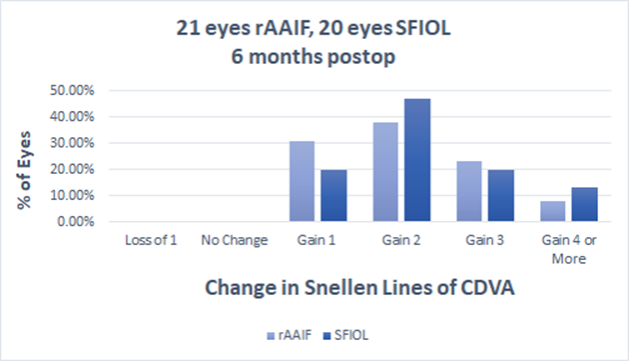
Standardized Graph of the Change in Corrected Distance Visual Acuity. Statistical analysis demonstrated similar results concerning CDVA in both groups. rAAIF = retropupillary Artisan Aphakia Iris-fixated IOL, SFIOL = scleral-fixated IOL

The mean preoperative CDVA for the rAAIF group was 0.10 ± 0.17, which improved to 0.23 ± 0.20 postoperatively. The average CDVA for the SFIOL group was 0.14 ± 0.16, which improved to 0.33 ± 0.25. Baseline CDVA was similar between the two groups as estimated by the Mann-Whitney test (*p*=0.30). The visual outcomes for CDVA at 6 months postoperatively were similar between the two groups (*p*=0.321) (**Fig. 2**). All patients were spectacle corrected and did not need to use alternative methods of correction, e.g. rigid gas permeable contact lenses.

Average postoperative astigmatism as assessed by a standard autorefractometer at the last follow-up 6 months postoperatively and at least 3 months after suture removal was 1.93 ± 1.38 in the rAAIF group and 1.58 ± 0.8 in the SFIOL group, which was not statistically different (*p*=0.22). 

Endothelial cell density was 1581.89 ± 556.57 preoperatively and declined to 1410.89 ± 558.48 postoperatively in the rAAIF group, and changed from 1995.75 ± 549.68 to 1803.13 ± 518.08 in the SFIOL group. Baseline ECD was similar between groups as shown by the Mann-Whitney test (*p*=0.19). The ECD loss in the two groups was also similar with *p*=0.89.

The validity of the questionnaire about patient satisfaction was confirmed using Cronbach’s alpha (*α*=0.904). Overall patient satisfaction score was 58.67±8.80 in the rAAIF and 56.69±11.50 in the SFIOL group, which was statistically similar (*p*=0.764). Comparisons between patient satisfaction for each parameter were estimated using the Mann-Whitney U test and are displayed in **Table 2**. 

**Table 2:** Comparison of patient satisfaction scores between the two groups for different parameters

Group/Question	*P*
Distance vision without glasses	0.79
Distance vision with glasses	0.94
Near vision without glasses	0.29
Near vision with glasses	0.81
Night vision	0.9
Halo	0.2
Starburst	0.69
Glare	0.2
Double vision	0.47
Dysphotopsias	0.30
Redness	0.03
Foreign body sensation	0.6
Ocular pain	0.9
Concern about the surgery	0.79
Information about the surgery	0.98

The only statistically significant difference between groups was observed in the self-reported redness with a mean score of 3.22 and 4.15 for the rAAIF and SFIOL groups, respectively, with 1 - easily visible redness (e.g. by other non-medical observers) and 5 - no redness at all.

## Discussion

Aphakia continues to be a surgical challenge in ophthalmological practice with different strategies available to correct the condition. Iris-claw IOLs, such as the Artisan Aphakia, have been used for more than 40 years with good results and constantly improving design. Recently, there has been growing evidence and preference of surgeons to implant the Artisan Aphakia Iris Fixation IOL in the posterior chamber. Posterior chamber implantation is preferred because of the better ECD, approximating physiological lens position, and more predictable refractive results [**5**-**7**]. In 2011, De Silva et al. [**5**] reported a series of 116 eyes of 104 patients, and in 2012, Gonnermann et al. [**7**] reported 137 eyes of 126 patients in whom Artisan lenses were placed in the posterior chamber; both reports offered pearls for improving technique and decreasing surgical time. Complications included wound dehiscence, IOL decentration, iris tissue dehiscence, increased intraocular pressure, hyphema, and cystoid macular edema. Al Dwairi compared retropupillary and anterior chamber Artisan IOL implantation and found that the visual outcomes were superior in the retropupillary group and the rate of complications was higher in the anterior chamber group [**8**]. 

In our study, the main complications observed were high postoperative IOP and corneal edema for both groups, as well as pupil ovalization in the rAAIF group. The complications reported in previous studies were less frequent or similar to those reported for anterior chamber or scleral-sutured lenses, which was also supported by our data. The complication rate between rAAIF and SFIOL was similar, albeit demonstrating some differences. As expected, we observed pupillary ovalization only in the AAIF group. Concerning common early postsurgical complications such as high IOP and corneal edema, no differences were observed between the two techniques. Since the follow-up was 6 months, longer-term complications could have been present, which we did not register, therefore possibly creating omission bias.

On the other hand, techniques for scleral fixation of IOL have also been improving to shorten procedure time and achieve better visual results as well as low complication rates. However, the main setback was the steep learning curve for mastering these challenging techniques. Teng and Zhang [**9**] compared AAIF implanted in the anterior chamber through a corneal incision and SFIOL and found that AAIF had shorter surgical time with similar long-term visual acuity and endothelial cell loss. Retropupillary AAIF requires longer surgical times than the anterior chamber, but it is still faster and easier than SFIOL. In our study, the decision for retropupillary implantation was based on previous scientific data by De Silva et al. [**5**] and Gonnermann et al. [**7**]. The patients in the SFIOL group of our study were younger. This could be explained by the assumption that the rate of IOL dislocation in the AAIF group would become higher with time since iris tissue could eventually erode considering life expectancy.

The comparable visual acuity in rAAIF and SFIOL could be explained by the similar effective lens position in both techniques. A meta-analysis by Jing et al. compared the two strategies for correcting aphakia - AAIF and SFIOL; however, in the study, the AAIF group comprised both anterior and posterior chamber implantation. The authors concluded that there was a preference among surgeons towards AAIF as it is less technically challenging and requires less surgical time. They found that SFIOL was preferred in more select cases [**4**].

Lajoie et al. [**3**] compared surgically induced astigmatism between AAIF implanted in the posterior or anterior chamber and found it to be similar with values of 1.67 D at 176° in the group with anterior versus 1.19 D at 11° in the group with posterior implantation. This corresponded to our results for postoperative astigmatism in the rAAIF (1.93 ± 1.38). However, in their study, the incision site was not specified. 

Although there are numerous studies on retropupillary iris-claw IOL implantation and scleral-fixated IOL separately, a comprehensive literature review did not reveal a comparison of the two techniques, especially concerning patient satisfaction. Despite the differences between the two types of surgery, they share a common goal - to correct aphakia. In our study, we found similar results about visual acuity, complication rates, endothelial cell loss, and patient satisfaction. The available reports focus on the implantation of phakic IOLs like the Artisan Phakic IOL for correction of myopia, which demonstrates excellent patient satisfaction [**10**]. In the available literature, usually, visual acuity is usually the only parameter considered for patient satisfaction in cases of aphakia correction and secondary IOL implantation. However, there are other aspects of the overall patient experience with any cataract surgery, as is well known. Regardless of the usually severe visual impairment in aphakia, patient satisfaction with secondary IOL implantation is still a factor that should be considered when choosing the most appropriate surgical strategy for each case. 

The main limitations of our study were the small sample size, as well as the non-blinded aspect of the surgery selection, which could be influenced by the incentive to use one IOL or another as per the surgeon’s preference.

## Conclusion

Retropupillary AAIF and SFIOL are both effective techniques for correcting aphakia with similar visual results, complication rates, ECD changes, and patient satisfaction. Despite the slightly higher preference towards rAAIF due to shorter surgical times, further research is needed to determine the factors for choosing one technique over the other. Careful individual preoperative assessment is required to have optimal results with either technique.

**Author contributions**

All authors contributed equally to this manuscript.

**Conflict of Interest Statement**

The authors state no conflict of interest.

**Informed Consent and Human and Animal Rights Statement**

Informed consent has been obtained from all individuals included in this study.

**Authorization for the use of human subjects**

Ethical approval: The research related to human use complies with all the relevant national regulations, and institutional policies, as per the tenets of the Helsinki Declaration, and has been approved by the review board of University Hospital Alexandrovska, Medical University Sofia, Sofia, Bulgaria (Entry 8352/24.11.2022, Protocol 04/15.05.2023).

**Acknowledgments**

None.

**Sources of Funding**

This work was not funded by any research or private grants.

**Disclosures**

None.
